# Prevalence, Haemolytic and Haemagglutination Activities and Antibiotic Susceptibility Profiles of *Campylobacter* spp. Isolated from Human Diarrhoeal Stools in Vhembe District, South Africa

**Published:** 2007-12

**Authors:** A. Samie, J. Ramalivhana, E.O. Igumbor, C.L. Obi

**Affiliations:** 1 Department of Microbiology, University of Venda, Thohoyandou 0950, South Africa; 2 Center for Global Health, University of Virginia, Charlottesville, VA 22908, USA; 3 College of Agriculture and Environmental Sciences, School of Agriculture and Life Sciences, Department of Life and Consumer Sciences, University of South Africa, Pretoria, South Africa

**Keywords:** Antibiotic resistance, *Campylobacter*, Diarrhoea, Enteritis, Gastroenteritis, Haemolysis, Haemagglutination, South Africa

## Abstract

*Campylobacter* species are increasingly being recognized as agents of gastroenteritis worldwide. However, data on the pathogenic characteristics of the organism isolated in rural communities in South Africa are lacking. In this study, the prevalence of *Campylobacter* spp. from diarrhoeal stools, haemolytic and haemagglutinating activities of the isolates, and antibiotic susceptibility profiles, including minimum inhibitory concentration (MIC) patterns to different antibiotics, were determined using the standard microbiological techniques. *Campylobacter* spp. were isolated from individuals of all age-groups; however, the infection rate was higher among individuals aged less than two years (30.4%). Of 115 *Campylobacter* strains isolated, polymerase chain reaction (PCR) analysis indicated that 98 (85%) were *C. jejuni*, while 17 (15%) were *C. coli*. Seventy-one (62%) of the strains showed haemolysis on human blood, and 80% agglutinated human blood, whereas 22.6% were β-lactamase-positive. Resistance to antimicrobials, such as erythromycin, ciprofloxacin, vancomycin, and fusidic acid, was high. Increased resistance to macrolide and quinolone antibiotics poses major risks for treatment failure. Haemolytic and haemagglutinating activities may be useful in preliminary characterization of pathogenic strains in settings where *Campylobacter*-associated infections are common.

## INTRODUCTION

During the past two decades, *Campylobacter* species have been more frequently identified as bacterial causes of human gastroenteritis in industrialized and developing countries (1). In the United States, for example, an estimated 2.4 million cases of human campylobacteriosis occur each year, and in England and Wales, the majority of general practitioner consultations, hospital admissions, and hospital bed days were due to *Campylobacter*-associated infections (2). In Africa, a few studies have indicated that campylobacteriosis is mostly common among children of young age. In Nigeria, for example, *Campylobacter jejuni* was found to be an important agent of diarrhoea in Ile-Ife and should be considered strongly in children with diarrhoea (3). Studies in Addis Ababa, Ethiopia, also indicated that *Campylobacter* spp. were important bacterial agents of diarrhoea in adults and children and should be considered routinely in the diagnosis of patients with diarrhoea (4). In Durban, South Africa, *Campylobacter* were found in 21% of diarrhoeal cases among children aged less than five years (5). In Venda, *Campylobacter* spp. were also isolated from 20% of stool samples tested from HIV-positive individuals (6); however, the pathogenic characteristics of these organisms have not been determined.

Results of recent studies indicated that *C. jejuni* and *C. coli* isolates from retail meat products exhibited a wide range of adherence and invasion abilities to human intestinal epithelial cells (T84 cells) almost as well as did *C. jejuni* 81-176 with a significant correlation between the adherence ability and the invasion ability of *Campylobacter* isolates (7). Whether *Campylobacter* organisms produce fimbriae that assist in adherence and colonization still remains uncertain, and despite their significance as human pathogens, little is known about the mechanisms they use to cause disease. Different methods for the verification of the presence of fimbriae, such as transmission electron microscopy, adherence assays, haemagglutination assays, and PCR, have been described (8).

Although diarrhoea due to *Campylobacter* can be self-limited, a small percentage of cases require medical intervention with erythromycin most commonly prescribed. However, patients with persistent diarrhoea or the immunosuppressed may require long-term antibiotic therapy (1). The evolution of multidrug-resistant strains also constitutes an important threat to the management of campylobacteriosis.

In the present study, haemagglutinating and haemolytic activities of *Campylobacter* spp. isolated from human diarrhoeic stools on human, sheep, chicken and pig red blood cells, antibiograms, including minimum inhibitory concentrations (MICs), were ascertained.

## MATERIALS AND METHODS

### Study sites and sample collection

The study was conducted in Vhembe district from November 2004 to May 2005. Diarrhoeic stool samples were obtained from patients attending four major public hospitals, namely Elim, Tshilidzini, Siloam, and Vhufuli. Samples were collected twice a week and were transported in cooler bags to the microbiology laboratory of the University of Venda for analysis. Demographic information, such as age and sex of patients, was obtained. Information on their HIV status was unknown.

### Isolation and identification of organisms

*Campylobacter* species were isolated using the standard microbiological methods: (a) The Cape Town protocol which involves the use of a filter paper on blood agar plate without antibiotics was used. Briefly, a suspension of faecal material was prepared in sterile saline. A 0.6 μ-m pore-size membrane filter (47 mm diameter) deposited on top of a Columbia blood agar plate supplemented with tryptose (Oxoid, England) was flooded with the stool emulsion. After 15 minutes, the filter was removed carefully preventing the suspension to pass to the media directly, so that only filtered material was inoculated on the agar (9). (b) A suspension of the faecal material in sterile saline was inoculated directly onto Skirrow's media (10) or (c) onto a charcoal-based media (mCCDA) using sterile swabs (10). All the media were purchased from Oxoid, England, and were prepared as indicated by the manufacturer. Suspected colonies that grew on all the media were confirmed by biochemical tests, including gram staining, oxidase, catalase, motility, and a *Campylobacter* haemagglutination kit ‘Campy Dry Spot’ (Oxoid, England) as recommended by the manufacturer.

### Testing of haemolytic activity

The haemolytic activity of *Campylobacter* isolates was tested on sheep, human, pig, and chicken blood agar. A single colony of each isolate was streaked across a blood agar plate using a sterile-inoculating wire loop. The inoculated blood agar plates were incubated at 37 °C for 24 hours in an environment with a gas mixture of 10% CO_2_, 5% H_2_, and 5% O_2_. After incubation, the haemolysis was determined by observing haemolytic zones around the bacterial growth on blood agar and scored as partial haemolysis or complete haemolysis. A maximum of six strains was tested on one plate.

### Testing of haemagglutination activity

The ability of *Campylobacter* isolates to agglutinate human, sheep, and chicken erythrocytes was tested as previously described (11). Briefly, blood cells were washed three times in a sterile phosphate-buffered saline, pH 7.4 (10 mM) (PBS) and a 3% (vol/vol) suspension was prepared in the same buffer immediately before use. Overnight cultures of *Campylobacter* strains, grown in Brain Heart Infusion broth (Oxoid, England), were centrifuged and washed twice in phosphate-buffered saline (PBS) and equilibrated to a McFarland standard no. 1. Haemagglutination tests were performed at room temperature by mixing 20 μL of erythrocyte suspension with 20 μL of bacterial suspension on a slide alongside a control suspension of erythrocytes and PBS. The slide was gently rocked by hand, and strains were considered positive if agglutination occurred within five minutes and negative if agglutination did not occur within this period. The same procedure was used with lysed red blood cells from the same animal species.

### Inhibition of haemagglutination

The inhibition of agglutination by D-mannose was tested as described above, except that 20 μL of blood suspension was added to 20 μL of a 1% (wt/vol) solution of D-mannose in PBS plus 20 μL of a suspension containing a McFarland no. 1 of bacteria in PBS. Reactions were compared with positive controls (20 μL of bacteria, 20 μL of PBS, and 20 μL of blood cells) and a negative control (40 μL of PBS and 20 μL of blood cells).

### β-lactamase production

β-lactamase production by *Campylobacter* isolates was detected by the nitrocefin test (12). Briefly, a 100 mL of liquid culture grown to an OD of 0.2-0.4, read at 600 nm, was centrifuged, and the pellet was washed twice in 10 mL of ice-cold phosphate buffer (pH 7.0) and resuspended in 1 mL of buffer. The suspension was sonicated and then centrifuged for 30 minutes at 10,000 g. The reaction was conducted in a 96-well microplate in which a 50-μL aliquot of the supernatant was added to 10 μL of nitrocefin. An immediate colour change from yellow to red was interpreted as positive for β-lactamase production.

### Determination of antibiotic susceptibility by the disc-diffusion test

Antibiotic susceptibility of *Campylobacter* isolates to 29 antibiotics was determined using the disc-diffusion technique. Information on the antibiotics and the disc (Oxoid, England) are shown in Table 2. Results were interpreted according to the guidelines of the National Committee for Clinical Laboratory Standards (now known as Clinical and Laboratory Standards Institute) for Enterobacteriaceae (13).

### Determination of minimum inhibitory concentrations by microdilution

The minimum inhibitory concentration (MIC) patterns of the isolates to 11 antibiotics were determined in microplates as previously described (13). Alternatively, the optical density was read in an ELISA reader at 495 nm. The antibiotics tested included gentamicin, nalidixic acid, ampicillin, penicillin G, erythromycin, tetracycline, kanamycin, ciprofloxacin, trimethoprim, D cycloserine, and chloramphenicol. All antibiotics (in powder form) were purchased from Sigma (Heidelberg, Germany).

### Species distribution of *Campylobacter* by PCR analysis

To confirm the species distribution of *Campylobacter* isolates, DNA was isolated from a fresh culture of each strain, and PCR primers, Hip 1a, and Hip 2b, specific to the hippuricase gene of *C. jejuni* were used (14), while the primer pairs—CC18F and CC519R—were used for identifying *C. coli* as previously described (15)

### Ethical considerations

The Research and Ethical Committee of the University of Venda, the Department of Health and Welfare, and the Department of Education in Polokwane, Limpopo province, South Africa, approved the study.

### Statistical analysis

To determine the significance of differences in infection rates, the SPSS software (version 10.1) was used. The differences were considered significant when the p value was less than 0.05.

## RESULTS

### Distribution of *Campylobacter*-associated infection in the study population

Of 565 stool samples collected from patients with diarrhoea, 290 were from females and 275 from males aged one month to 76 years with a median of 37 years. Of 69 individuals infected due to *Campylobacter*, the most commonly infected were 21 individuals (30.4%) aged 0-2 year(s). The infection rate was higher among females (21.7%; 63/290) than among males (18.9%; 52/275). However, the difference was not statistically significant (χ^2^=0.690, p=0.406, odds ratio=0.840, 95% confidence interval [CI] 0.557-1.267) even when each age-group was considered separately. Table 1 indicates the distribution of the study population by age-group and sex and also by the infection rate of *Campylobacter* spp.

**Table 1:** Distribution of study population by age-group and sex and also by rate of isolation of *Campylobacter* spp. in diarrhoeal stools from hospitals in Vhembe district, South Africa

Age-group (years)	Males		Females		Total	
	No. (%) of individuals	*Campylobacter*-positive (%)	No. (%) of individuals	*Campylobacter*-positive (%)	No. (%) of individuals	*Campylobacter*-positive (%)

0-2	33 (12)	9 (27.3)	36 (12.4)	12 (33.3)	69 (12.2)	21 (30.4)
3-5	22 (8)	2 (9)	25 (8.6)	6 (24)	47 (8.3)	8 (17)
6-9	30 (11)	6 (20)	21 (7.2)	4 (19)	51 (9)	10 (19.6)
10-19	34 (12.4)	3 (8.8)	38 (13.1)	5 (13.1)	72 (12.7)	8 (11.1)
20-29	46 (16.7)	11 (23.9)	52 (18)	10 (19.2)	98 (17.3)	21 (21.4)
30-39	47 (17)	12 (25.5)	48 (16.6)	8 (16.7)	95 (16.8)	20 (21)
40-49	41 (15)	8 (19.5)	42 (14.5)	12 (28.6)	83 (14.7)	20 (24.1)
50-60	12 (4.4)	1 (8.3)	16 (5.5)	4 (25)	28 (5)	5 (17.8)
>60	10 (3.6)	0 (0)	12 (4.1)	2 (16.7)	22 (3.9)	2 (9)
Total	275 (48.7)	52 (18.9)	290 (51.3)	63 (21.7)	565 (100)	115 (20.3)

### Haemolytic and haemagglutination activities and β-lactamase production of *Campylobacter* isolates

Seventy-one (61%) of the *Campylobacter* isolates showed haemolytic activity on human red blood cells, and these comprised 18 (15.7%) with partial haemolysis and 53 (46.1%) with complete haemolysis. Pig blood was less sensitive to haemolysis by *Campylobacter* spp. with 17.4% showing complete haemolysis, whereas 42% of the isolates were haemolytic on chicken and sheep red blood cells.

Two types of haemagglutination were observed: one that was inhibited by mannose–termed type 1 and one that was not inhibited by mannose–termed type 3 (16). About 92 (80%) of the isolates demonstrated haemagglutinating activity on chicken and human red blood cells, 21% of which were inhibited by mannose, and 59% were not inhibited by mannose. Sheep blood was resistant to haemagglutination.

Twenty-six (22.6%) of 115 *Campylobacter* spp. isolated from human diarrhoeal stools were beta-lactamase-positive.

### Antibiotic susceptibility profiles and distribution of MICs of different antibiotics

Table 2 shows the results of antibiotic susceptibility testing of the *Campylobacter* isolates to the 29 antibiotics studied, the antibiotic content of the discs, the code name of the antibiotics, and the resistance breakpoints used. The patterns of MICs of 11 antibiotics on the *Campylobacter* isolates were determined with concentrations varying from 0.25 μg/mL to 128 μg/mL as indicated on Table 2.

**Table 2:** Antibiotic susceptibility profiles of *Campylobacter* spp. isolated from diarrhoeal stool samples in Vhembe district

Class of antibiotics	ATB	Code	Con (μg)	RBP (mm)	No. resistant (%)	MIC range

Penicillins	Penicillin G	PG	10	≤14	115 (100)	≥128 μg/mL–8 μg/mL
	Amoxicillin	A	10	≤13	115 (100)	ND
	Cloxacillin	CX	5	≤13	103 (89.6)	ND
	Ampicillin	AP	10	≤13	94 (81.2)	≥128 μg/mL–8 μg/mL
Cephalosporines	Cephazoline 1^st^	CZ	30	≤14	77 (67)	ND
	Cefuroxime 2^nd^	CXM	30	≤14	87 (76)	ND
	Cefoxitin 2^nd^	FOX	30	≤14	36 (31.4)	ND
	Cefotaxime 3^rd^	CTX	30	≤14	33 (28.7)	ND
	Ceftriaxone 3^rd^	CRO	30	≤14	8 (7)	ND
	Cefepime 4^th^	CPM	30	≤14	9 (8)	ND
Carbapenems	Meropenem	MEM	10	≤13	11 (9.6)	ND
	Imipenem	IMI	10	≤13	13 (11.3)	ND
Quinolones	Nalidixic acid	NA	30	≤13	59 (51.3)	128 μg/mL–1 μg/mL
	Ciprofloxacin	CIP	5	≤15	15 (13)	32 μg/mL–0.25 μg/mL
	Lomefloxacin	Lom	10	≤13	17 (14.8)	ND
Aminoglycosides	Gentamicin	GM	10	≤12	20 (17.3)	32 μg/mL–0.25 μg/mL
	Amikacin	AK	30	≤14	20 (17.4)	ND
	Kanamycin	K	30	≤14	21 (18.3)	32 μg/mL–0.25 μg/mL
Tetracyclines	Tetracycline	T	30	≤14	31 (27)	64 μg/mL–0.25 μg/mL
	Doxycycline	DXT	30	≤12	46 (40)	ND
Macrolides	Erythromycin	E	15	≤13	61 (53)	64 μg/mL–0.5 μg/mL
Glycopeptide	Vancomycin	VA	30	≤14	115 (100)	ND
Others	Fusidic acid	FC	10	≤14	113 (98)	ND
	Rifampicin	RP	5	≤13	86 (75)	ND
	Chloramphenicol	C	30	≤12	24 (21)	128 μg/mL–0.5 μg/mL
	Augmentin	AUG	30	≤14	96 (83)	ND
	Nitrofurantoin	NI	300	≤14	76 (66.1)	ND
	Novobiocin	NO	5	≤14	115 (100)	ND
	Co-trimoxazole	TS	20	≤13	42 (36.5)	ND
	Trimethoprim	TRP	-	-	ND	≥128 μg/mL–64 μg/mL
	D-cycloserine	DCS	-	-	ND	4 μg/mL–0.25 μg/mL

ATB=Antibiotics; Con=Antibiotic content of the disc; MIC=Minimum inhibitory concentration; ND=Not done; RBP=Resistance break-point

### Confirmation of species distribution by PCR

Of all the 115 isolates tested for both *C. jejuni* and *C. coli* by PCR, 98 (85%) were *C. jejuni*, and 17 (15%) were *C. coli*. The figure shows the electrophoresis gels of the PCR products for *C. jejuni* and *C. coli*.

### DISCUSSION

The aim of this study was to isolate *Campylobacter* spp. from diarrhoeal stool samples obtained from patients attending major hospital centres in Vhembe district and to determine their characteristics in terms of haemolysis, haemagglutination, ß-lactamase production, and antibiotic susceptibility profiles. We found a higher rate of infection among children aged less than two years, similar to the results obtained from other studies, such as those undertaken in Barbados that indicated the highest rate of isolation in children aged 1-4 year(s) (40.8%) (17). Results of recent studies in Kenya and Spain also indicated a higher prevalence of *Campylobacter* spp. among children aged less than five years (18,19). It is, thus, important that control programmes targeting the elimination of infections due to *Campylobacter* consider children as a priority since they might be more vulnerable to infections than adults.

Haemolysis has been determined as a pathogenic factor in *Campylobacter* spp. and is associated with inflammatory *Campylobacter* strains (20). We found that 62% of the *Campylobacter* strains were haemolytic on human blood. This compares with results of previous studies which indicated that 17 (71%) of 24 human and 61 (63%) of 97 pig isolates showed haemolytic activity on sheep blood (21). It has been suggested that the adherence capacity and cytotoxicity could be used as virulence markers and for predicting the inflammatory or secretory nature of *C. jejuni*-induced diarrhoea. (20). In the present study, we could not differentiate between different types of diarrhoea; however, the high rate of haemolytic strains on human blood may indicate the inflammatory characteristics of these strains. Profound haemolytic activities against human blood as opposed to pig blood were noted. Hossain *et al.* demonstrated that *Campylobacter* strains produced one or more haemolysin(s) with maximum activity against rabbit erythrocytes and minimal activity against chicken erythrocytes (22). This also indicates the different degrees of susceptibility of different animal species to *Campylobacter*-associated infections and the increased susceptibility of humans to these *Campylobacter* strains.

The major therapeutic intervention for all individuals with diarrhoea consists of fluid and electrolyte therapy. However, the use of antibiotics may help shorten the duration of diarrhoea and speed recovery. In the case of *Campylobacter*, the drugs of choice include macrolide and fluoroquinolones. However, resistance to these and other antibiotics has been reported around the world. In the Venda region, the site of our study, the resistance of *Campylobacter* spp. to erythromycin has been increasing steadily. In 2002, Obi and Bessong reported a resistance level of 25% (6), and in 2004, Obi *et al.* reported a resistance level of 35% (23). In the present study, we found a resistance level of 53%. The same was also noted for resistance to ciprofloxacin (8%) (6)—4% in 2004 (23), and we found 13% resistance in the present study. Resistance to gentamicin rose from 8% in 2002 to 12% in 2004, and it was 17.3% in the present study. Increasing resistance constitutes a great risk of treatment failure and underscores the importance of monitoring antibiotics and the quest for alternative strategies to treat bacterial infections. Rates of similar resistance have also been reported from other parts of the world. In England and Wales, over half (55%) of *Campylobacter*-associated infections acquired abroad (Spain, Portugal, or Cyprus) were resistant to ciprofloxacin compared to 10% of UK-acquired strains (24). In Ireland, resistance among human *Campylobacter* isolates was 6.4%, 12%, and 13% to erythromycin, ciprofloxacin, and tetracycline respectively, with minor differences between *C. jejuni* and *C. coli* strains (1). In Senegal, Cardinale *et al.* observed high rates of quinolone-resistance for *C. jejuni* (43.4%) and *C. coli* (48.6%) isolates obtained from poultry (25). However, the antibiotic resistance of *Campylobacter* isolates in meat products has not been studied in the Venda region of South Africa, and conducting similar studies on poultry would enhance our understanding of their possible role in antibiotic resistance. When antimicrobial therapy is indicated, the selection of a specific agent should be made based upon the susceptibility patterns of the pathogen or information on local susceptibility patterns (26).

**Fig.**
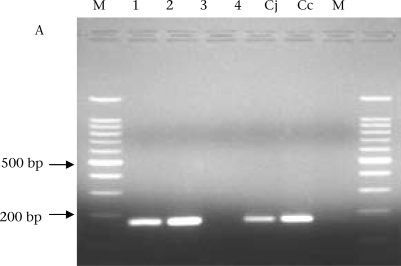

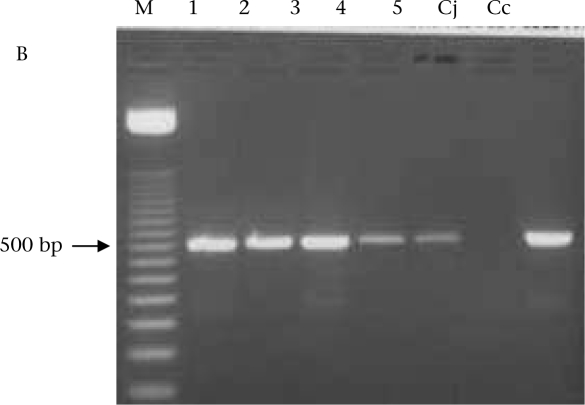
PCR confirmation of *Campylobacter* isolates. A: Hippuricase gene PCR products for confirmation of *C. jejuni*; M=DNA ladder 1, 2, and 4 are isolates positive for *C. jejuni*, 3=Negative control, Cj=*C. jejuni* (ATCC 33560) and Cc=*C. coli* (ATCC 33559) used as positive controls. B: PCR products for confirmation of *C. coli*: 1, 2, 3, 4, and 5 are strains positive for *C. coli*

PCR=Polymerase chain reaction

Finally, the study has provided baseline data on the prevalence, antibiograms, and some pathogenic factors among *Campylobacter* isolates and is, therefore, of clinico-epidemiological significance.
